# Dynamics of Germination Behaviour, Protein Secondary Structure, Technofunctional Properties, Antinutrients, Antioxidant Capacity and Mineral Elements in Germinated Dhaincha

**DOI:** 10.17113/ftb.59.02.21.6922

**Published:** 2021-06

**Authors:** Savita Sharma, Prashant Sahni

**Affiliations:** Department of Food Science and Technology, Punjab Agricultural University, Ludhiana-141004 (Punjab), India

**Keywords:** processed dhaincha, bioactive components, functional properties, FTIR

## Abstract

**Research background:**

Dhaincha (*Sesbania aculeata*) is a forage legume primarily used for green manuring and animal feed. Good nutritional profile of dhaincha makes it a potential alternative legume in human nutrition. However, the presence of high amount of antinutrients poses a problem in its utilisation for food applications. The present investigation intends to germinate dhaincha seeds at different time-temperature regimes and to evaluate the process of germination to ascertain optimal conditions and improve its potential for utilisation.

**Experimental approach:**

Dhaincha seeds were germinated at 24, 28 and 32 °C for 24, 48 and 72 h. Germination characteristics and germination loss, spectral characteristics, technofunctionality, antinutrients, bioactive constituents, antioxidant capacity and mineral element content of germinated dhaincha were evaluated. Optimal balance of technobiofunctionality of germinated dhaincha seeds was validated by principal component analysis.

**Results and conclusions:**

Sprout length and germination loss increased with the higher germination temperature and prolonged germination time. Seeds showed similar germination rate at 28 and 32 °C and it was markedly higher than at 24 °C. Germination for 24 h resulted in mild conformational changes in the secondary structure of proteins, whereas germination for 48 and 72 h exhibited major conformational changes in the β-sheets, resulting in the improvement in the hydration and foaming properties. Progression of germination (72 h) caused the decrease of tannin (24.47%), phytic acid (16.38%) and saponin (24.58%) mass fractions, and of trypsin inhibitor (40.33%) and lectin activity (62.50%). Slight decrease of DPPH˙ (3.7%) and ABTS˙^+^ (18.5%) values was also observed, whereas total flavonoid content (36.14%) and metal chelating activity (26.76%) increased. Total phenolics, FRAP, and reducing power decreased after 24 h, followed by a gradual increase. Zinc extractability increased drastically with germination. Germination at 28 °C for 72 h resulted in higher reduction of antinutrients with optimal retention of antioxidant activity and better functional characteristics, as validated by principal component analysis.

**Novelty and scientific contribution:**

Dhaincha is an unknown crop in Europe, and even in Asia it is predominantly used as green manure and animal feed. This research demonstrated that the intervention in germination can transform dhaincha into a promising crop for food industry. Germinated dhaincha exhibited enhanced technobiofunctionality for utilisation in various food formulations.

## INTRODUCTION

Germination of seed legumes is a natural, economical, and widely practiced non-thermal food processing technique employed to enhance their potential for utilisation. It induces changes in the biochemical characteristics of legumes as a result of degradation of reserve material during respiration and sprouting, as well as due to the biogenesis of new cell constituents and secondary metabolites that improves the antioxidant capacity of legumes (*1*, *2*). Furthermore, the reduction in the antinutrients like phytic acid, lectins, saponins, trypsin inhibitor and tannins due to germination improves the nutritive value of germinated legumes (*3*-*6*). Besides altering the biochemical characteristics of legumes, germination also causes modification in the structural characteristics and associated functional behaviour of macromolecules to impart enhanced technofunctionality to germinated flour for better utilisation in the food formulations (*7*, *8*). Evaluation of the dynamics of various characteristics of legumes during germination allows the understanding of technobiofunctional nuances to ascertain the best regime of germination for enhancement in the functionality of legumes.

Dhaincha (*Sesbania aculeata* or *S. bispinosa*) is a rapidly growing non-conventional legume crop, which is well suited for adverse soil and climatic conditions and shows tolerance to disease and pest infestation (*9*). It is extensively found in many tropical countries of Asia and Africa, with seed yield of 1.5 t/ha and minimum seed yield of 1 t/ha under Indian farm-scale conditions (*10*). Dhaincha is mainly utilised for green manuring and animal feeding (*11*). However, some Indian tribal sects (Katkari and Gond) consume cooked dhaincha seeds (*12*). Few studies have reported the nutritional composition of dhaincha and the reluctance to consume it due to high amount of associated antinutrients and galactomannans that tend to reduce its nutritive value (*10*, *12*, *13*). Mehta *et al.* (*14*) have demonstrated antioxidant and anticancer activity of dhaincha seeds. High protein content, good amino acid composition, essential polyunsaturated fatty acids and associated total phenolics make it an alternative legume for exploration in food applications (*10*, *14*). However, no work has been carried out on the germination of dhaincha seeds to improve its potential for food use. Thus, the present investigation intends to assess the dynamics of germination behaviour, technofunctionality, antinutritional factors, bioactive components, antioxidant capacity and mineral elements in germinated dhaincha to evaluate the effect of germination on the overall functionality of dhaincha flour, validated using principal component analysis.

## MATERIALS AND METHODS

### Material

Clean and healthy dhaincha seeds (Punjab dhaincha 1) were taken from Punjab Agricultural University, Ludhiana (India). Seeds were stored in airtight PET jars under cool and dry conditions at 4 °C. Reagents used in the study were of analytical grade and were procured from Sisco Research Laboratories Pvt. Ltd., Modern Instruments and Chemicals, Ludhiana, Punjab (India). All the chemical standards used in the study were procured from Sigma-Aldrich Chemicals Pvt. Ltd., Bangalore, Karnataka (India).

### Steeping behaviour

Dhaincha seeds were disinfected by steeping in 0.1% sodium hypochlorite solution for half an hour, followed by rinsing with distilled water. Steeping behaviour of dhaincha seeds was evaluated by soaking 100 g seeds in distilled water (1:10 *m*/*V*) at different temperatures (24, 28 and 32 °C), noting the moisture content after every hour and plotting mass fraction (in %) of moisture against steeping time (h) (*15*).

### Germination of dhaincha

Dhaincha seeds were steeped in distilled water at 24, 28 and 32 °C. Hydrated seeds were spread on wetted double-layer muslin cloth. Temperature of 24, 28 and 32 °C and time of 24, 48 and 72 h were employed for the germination of dhaincha seeds. Seeds were germinated under different conditions in an incubator (Narang Scientific Works, New Delhi, India) kept at relative humidity of 90-95%. The muslin cloth was kept moist throughout the germination by wetting it with distilled water at regular intervals. Germinated seeds were dried at 50 °C in hot air oven (Shivam Instruments, New Delhi, India) up to *w*(moisture)=8% (*8*).

### Germination characteristics

Germination characteristics were evaluated by germinating 100 healthy dhaincha seeds (*16*). Sprout length was measured with digital vernier calliper. Germination capacity (%) was evaluated by counting the number of germinated seeds out of total seeds. Germination rate was evaluated by determining the number of seeds germinated at 24 (G1), 48 (G2) and 72 (G3) h and calculated according to the following equation:

Germination rate=(*N*_G1_·100+*N*_G2_·50+*N*_G3_·33.3)/*N*_GTotal_ /1/

### Germination loss

Vegetative portion was removed from sprouted seeds by rubbing them off gently. The loss of dry matter was determined on the basis of *m*(kernel)_initial_=1000 according to the following equation:

*N*(kernel)=((*m*(kernel)_initial_-*m*(kernel)_final_)/*m*(kernel)_initial_)∙100 /2/

Germination loss was evaluated as follows (*17*):


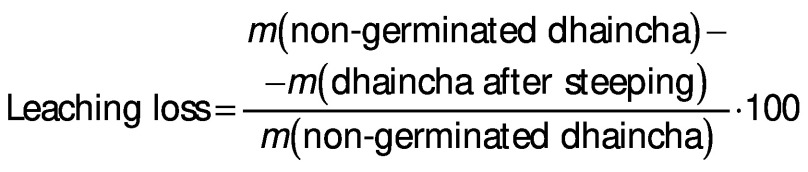



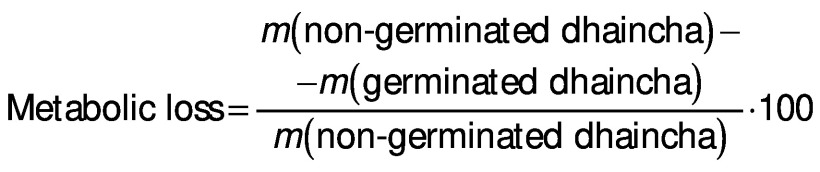



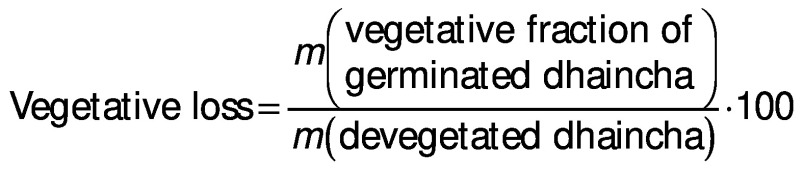


### Preparation of the flour

Seeds were milled in cyclotec mill (Newport Scientific, Warriewood, NSW, Australia). The prepared flour was sifted using 60 mesh sieve and stored in airtight PET jars under cool and dry conditions (4 °C).

### Spectral characteristics

Spectral characteristics of ungerminated and germinated dhaincha flour were recorded to evaluate the protein secondary structure using attenuated total reflection Fourier transform infrared (ATR-FTIR) spectrometer (Nicolet 67000; ThermoFisher Scientific, Madison, WI, USA). Spectra were recorded in the range 400-4000 cm^-1^.

### Functional properties

To evaluate bulk density, 20 g dhaincha flour were placed in measuring cylinder, gently tapped, measuring the volume in g/cm^3^ (*8*).

For the measurement of water absorption capacity, 3 g dhaincha flour were placed in a previously weighed centrifugation tube and 30 mL distilled water were added. The flour was allowed to absorb water for 30 min with gentle tapping after every 10 min, centrifuged at 2000×*g* in a centrifuge (Laby Instrument Industry, Ambala, Haryana, India) and then the water was decanted. The quantity of water absorbed by flour was expressed in g/g (*8*).

For the measurement of oil absorption capacity, 3 g dhaincha flour were placed in a previously weighed centrifugation tube and 30 mL oil were added. The flour was allowed to absorb oil for 30 min with gentle tapping after every 10 min, centrifuged at 2000×*g* in a centrifuge (Laby Instrument Industry) and then the oil was decanted. The quantity of oil absorbed by flour was expressed in g/g (*8*).

Swelling capacity was measured by taking 500 mg dhaincha flour in a previously weighed centrifugation tube and 15 mL distilled water were added to it. The centrifuge tube was covered and kept in a water bath at 90 °C, followed by cooling and centrifugation at 2000×*g* (Laby Instrument Industry) and decanting the water (*8*).

For the measurement of water solubility index and leaching loss, the decanted water obtained after evaluating water absorption and swelling capacity, respectively, was taken in a previously weighed Petri dish and dried at 100 °C. The mass of Petri dish was noted after drying to determine the mass of solid leached in the water. Water solubility index and leaching loss were expressed in precentage (*8*).

For the evaluation of emulsification capacity, 2 g dhaincha flour were taken in the centrifugation tube and 20 mL distilled water and 20 mL oil were added to it. The mixture was emulsified by vigorous shaking, followed by centrifugation at 3500×*g* for 10 min, and the height of the emulsion layer was measured. The emulsion stability was determined by heating the emulsion in a water bath (WBC012; LABQUEST, Borosil, Pune, India) at 80 °C for 30 min, followed by cooling and centrifugation under the aforesaid conditions and measuring the reduction in the height of the emulsion layer (*8*).

To evaluate foaming capacity, 2 g dhiancha flour were taken and 100 mL distilled water were added. The suspension was blended for 1 min using a blender (Kalsi Company, Ambala, India), the content was transferred to a 250-mL measuring cylinder and the volume of the developed foam was measured and expressed in percentage in relation to initial volume of the suspension (*8*). Foam characteristics were assessed visually and recorded as foam appearance (*18*). Foam stability was evaluated every 10 min for 1 h by recording the reduction in the foam volume (*8*).

Dispersibility was determined by taking 10 g dhaincha flour in a 200-mL measuring cylinder, followed by the addition of distilled water up to 100 mL mark. Flour and distilled water were mixed well and left undisturbed for 3 h. The volume of the settled flour particles was measured, subtracted from 100 and expressed as percentage of dispersibility. Gel consistency was measured by dispensing 200 mg dhaincha flour in 200 µL ethanol, and 3 mL distilled water or 0.1 M acetic acid were added to measure gel consistency in water and acid respectively. Flour suspensions were heated in boiling water bath for 8 min, followed by cooling and placing them on the levelled surface for 1 h. The distance travelled by the gel in the test tubes was measured in cm (*8*).

To evaluate swelling index, 1 g dhaincha flour was taken in a measuring cylinder. The initial volume of the flour was noted, 10 mL distilled water were added and the mixture was undisturbed for an hour. Then the volume of the swelled flour was measured and expressed as swelling index by taking the ratio of the volume of the swelled flour and the initial volume of the flour (*19*).

### Gelation behaviour

Dispersions of dhaincha flour (2-30% *m/V*) were prepared in distilled water in a test tube, vortexed well and heated for 1 h using boiling water bath. The tubes were subsequently cooled under running water and kept at 4 °C for 1 h (*8*). The test tubes were inverted to observe the flow of the gel from the test tubes. When the gel did not fall from the tube it was denoted by +, whereas slight flow was considered partial gelation and was denoted by ±, and no gelation was denoted by -. Gel was visually characterised and recorded as gel appearance (*18*).

### Estimation of antinutrients

Extraction of tannins was carried by employing *φ*(methanol)=10%. Tannins were evaluated colorimetrically by adding 1 mL extract, mixing it with 75 mL distilled water and 5 mL Folin-Denis reagent, followed by the addition of 10 mL saturated sodium carbonate solution and making a total volume of 100 mL with distilled water. Absorbance was measured at 700 nm using spectrophotometer (LMSP-V325; Labman Scientific Instruments Pvt. Ltd, Tamil Nadu, India) and the results were compared to the tannic acid standard solutions and expressed in mg/g (*20*).

Phytic acid was extracted with 0.5 M HNO_3_ and then 1.4 mL distilled water were added to 0.5 mL of extract, followed by the addition of 1 mL ammonium iron(III) sulphate solution (containing 50 µg iron). The contents were transferred to the test tube and boiled in a water bath for 20 min and cooled subsequently. A volume of 5 mL amyl alcohol was added to the content of the test tube, followed by the addition of 0.1 mL of 10% NH_4_SCN solution, centrifuged at 2000×*g*, alcoholic layer was separated and the absorbance was measured (LMSP-V325; Labman Scientific Instruments) at 465 nm (*20*).

Saponins were extracted with acetone for 24 h, followed by the removal of acetone, then 5 mL methanol were added and the mixture was extracted with the aforesaid procedure. The extract was then diluted with methanol and made up to the volume of 15 mL. Saponins were quantified by taking 1 mL of sample extract and evaporating the solvent in boiling water bath. After the removal of the solvent, 2 mL ethyl acetate were added and mixed well. A volume of 1 mL reagent (5 µL anisaldehyde+995 µL ethyl acetate), followed by 1 mL of concentrated H_2_SO_4_ were added to the test tube, mixed and allowed to stand at room temperature for 10 min. Absorbance was measured at 430 nm and saponin mass fraction was expressed in mg diosgenin equivalent (DE) per g of flour (*21*).

For determination of trypsin inhibitor activity, 1 g flour sample was extracted with 0.01 M phosphate buffer (pH=7.5) and stirred for 1 h at room temperature, then centrifuged at 2000×*g* for 30 min and supernatant containing trypsin was collected. A volume of 50 µL trypsin extract was taken, 50 µL bovine trypsin and 100 μL 0.01 M Tris HCl buffer (pH=7.5) were added and incubated for 10 min at 37 °C. Trypsin inhibitor activity of the extract was evaluated using *N*-α-benzoyl-dl-arginine-*p*-nitroanilide as a substrate by incubating at 37 °C for 10 min. Reaction was stopped using 200 µL of 30% acetic acid and absorbance was measured at 410 nm. The activity of trypsin inhibitor was expressed in inhibition unit (IU) per mg protein and 1 IU corresponded to the increase of absorbance by 0.01 per 10 mL (*21*).

Lectins were extracted from dhaincha flour using phosphate-buffered saline at pH=7.2. A blood suspension of 2% was prepared by mixing the trypsinised rabbit blood in normal saline (0.9%). Agglutination of blood suspension was expressed as haemagglutinin unit per g of flour. One U corresponds to the reciprocal value of the agglutination at the highest dilution (*22*).

### Bioactive constituents

Extraction was carried out in *φ*(methanol)=80%. A mass of 2 g sample was mixed with 20 mL acidified methanol, refluxed for 2 h and supernatant was collected by centrifugation at 2000×*g*. Residue was again refluxed for 2 h with 20 mL acidified methanol, the obtained supernatant was pooled with the previous supernatant and made to 50 mL volume with acidified methanol. For the estimation of total phenolics, 200 µL methanolic extract were made up to 1 mL with distilled water and 5 mL 10% (*V*/*V*) Folin-Ciocalteu reagent were added, followed by the addition of 4 mL 7.5 % Na_2_CO_3_ after 5 min, then the mixture was vortexed and allowed to stand in the dark for 15 min. The absorbance was measured at 765 nm. For evaluation of total flavonoids, the volume of 0.5 mL extract was made up to 2 mL by adding methanol, and then 0.1 mL 10% aluminium chloride, 0.1 mL potassium acetate solution and 2.8 mL distilled water were added. The mixture was vortexed well at 1200 rpm and the absorbance was measured at 415 nm. Total phenolic (*23*) and total flavonoid contents (*24*) were expressed in mg of gallic acid equivalents (GAE) and quercetin equivalents (QE) per g of flour respectively.

### Antioxidant capacity assays

The extract prepared above for the estimation of bioactive constituents was used for the evaluation of DPPH˙ and ABTS˙^+^ scavenging activity, iron(III) reducing antioxidant power (FRAP) and reducing power.

To determine DPPH˙ scavenging activity, 1 mL Tris buffer was added to 1 mL methanolic extract in a test tube, followed by the addition of 2 mL 2,2-diphenyl-1-picryl hydrazyl (DPPH). The test tubes were incubated for 30 min in the dark. Absorbance was measured at 517 nm (*24*). For the determination of ABTS˙^+^ scavenging activity, 7.4 mM ABTS˙^+^ solution and 2.6 mM potassium persulfate solution were mixed in equal quantities and allowed to react for 12 h at room temperature in the dark. The solution was then diluted by mixing 1 mL ABTS˙^+^ solution with 60 mL methanol to obtain an absorbance of (1.10±0.02) AU at 734 nm. To 3 mL ABTS test reagent, 1 mL sample extract was added and incubated to react in dark for 2 h and the absorbance was measured at 734 nm (*25*). DPPH˙ and ABTS˙^+^ scavenging activities were expressed in µmol Trolox equivalents (TE) per 100 g of flour.

For the estimation of FRAP, stock solutions of 300 mM acetate buffer, 10 mM 2,4,6-tripyridyl*-s-*triazine (TPTZ) solution in 40 mM HCl, and 20 mM FeCl_3_·6H_2_O solution were prepared. The fresh working solution was prepared by mixing acetate buffer, TPTZ solution and FeCl_3_·6H_2_O solution in 10:1:1 volume ratio, respectively. A volume of 0.6 mL sample extract was taken, 4.5 mL FRAP solution were added and kept in the dark for 30 min. Then, the colour intensity was measured at 593 nm. FRAP was expressed in µmol TE per g of flour (*24*).

For determination of reducing power, 2.5 mL phosphate buffer (pH=6.6) and 2.5 mL of 1% potassium iron(III) cyanide were added to 1 mL extract, followed by incubation for 20 min. A volume of 2.5 mL of 10% trichloroacetic acid was added and the solution was centrifuged at 3000×*g* for 20 min. A volume of 2.5 mL of the obtained supernatant was mixed with 2.5 mL water and 0.5 mL FeCl_3_ (0.1%). Absorbance was measured at 700 nm and expressed in mg ascorbic acid equivalents (AAE) per g of flour (*26*). For determination of metal chelating activity, 0.5 g flour was extracted with *φ*(methanol)=75% using orbital shaker for 2 h. The sample was centrifuged at 2000×*g* and the volume was made to 50 mL with 75% methanol. A volume of 1 mL methanolic extract was taken in a test tube and 1 mL 0.1 mM FeSo_4_ was added, followed by the addition of 1 mL of 0.25 mM ferrozine solution and incubation for 10 min. Absorbance was measured at 562 nm and the results were expressed in mmol ethylenediaminetetraacetic acid (EDTA) equivalents per 100 g of flour (*27*).

### Mineral elements

A mass of 1 g dhaincha flour sample was taken and digested by microwave-assisted digestion using HNO_3_ and HClO_4_ in a ratio 3:1. The digested sample was diluted with deionised water up to 50 mL volume, filtered and macro- and microminerals were measured by inductively coupled plasma-mass spectrometry (ICP-MS) (X-Series2; ThermoFisher Scientific). Mineral elements were expressed in mg/kg (*28*).

### Statistical analysis

Ten replications were taken for evaluating sprout length; five replications were taken for germination rate and capacity, mass of a thousand kernels and germination loss, and all the other analyses were carried out in triplicate. Analysis of variance (ANOVA) followed by *post hoc* Tukey’s test was performed to evaluate the statistical significance (p<0.05) using SPSS software, v. 22.0 (*29*). Pearson’s correlation and principal component analysis (PCA) were performed using Statistica v. 12 (*30*) to find correlation between the bioactive constituents and the antioxidant capacity, and to evaluate the dynamics of techno-biofunctionality of germinating dhaincha.

## RESULTS AND DISCUSSION

### Hydration behaviour

**Fig. 1:**
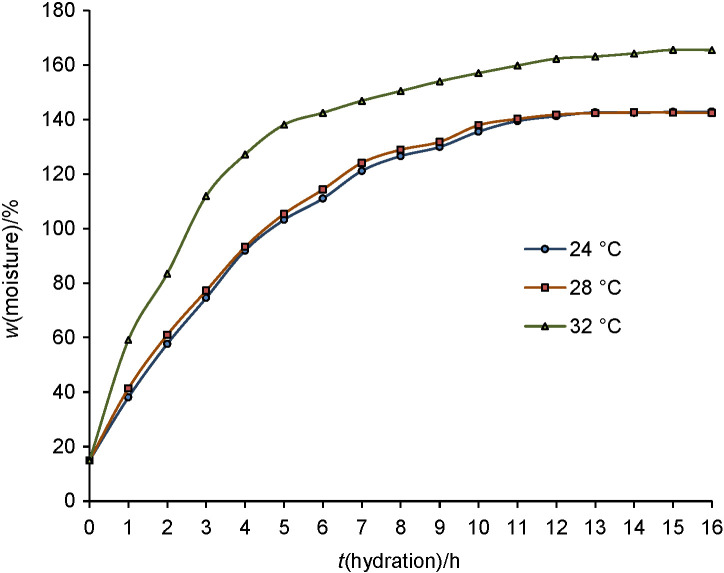
Hydration isotherms of dhiaincha seeds at different temperatures. Values are expressed as mean±standard deviation on dry mass basis (*N*=3)

Hydration isotherms of dhaincha seeds at different temperatures is presented in Fig. 1. Optimum steeping time is important to properly hydrate the seeds to allow the activation of enzymes (*31*). Rapid uptake of water was observed during the first 5 h of soaking, followed by gradual water uptake up to 12 h. Maximum hydration was observed after 12 h in seeds soaked at 24 and 28 °C in contrast to maximum hydration time of 16 h for seeds soaked at 32 °C. Furthermore, seeds soaked at 32 °C absorbed more water than the seeds steeped at 24 and 28 °C. The extent and time required for the imbibition of water is dictated by the activation energy required for the diffusion of moisture based on the composition of grain, temperature of steeping and amount of water taken for steeping. Seeds tend to show higher diffusivity at initial period of soaking with higher hydration as a function of temperature due to increase in the driving force and reduction in the resistivity of grain to the diffusion (*15*). Montanuci *et al.* (*15*) and Malleshi and Desikachar (*17*) reported high water uptake at elevated temperature of soaking of barley and finger millet, respectively. Seeds were soaked for 12 and 15 h for germination at 24 and 28 °C, and 32 °C, respectively.

### Sprouting characteristics

Maximum germination rate at optimum temperature facilitates the activation of metabolic processes due to enhanced enzymatic activity during seed germination (*31*, *32*). At 28 and 32 °C, germination rate was high with similar values, unlike at 24 °C (Table 1). Germination capacity of seeds increased gradually with the increase in the germination time. However, no significant difference was observed in the germination capacity at 28 and 32 °C. Higher germination temperature and time resulted in the increase in the sprout length. Heidari *et al.* (*32*) and Devi *et al.* (*33*) also observed similar trend of increase in the sprout length with higher germination temperature and prolonged germination time.

**Table 1:** Germination characteristics at different temperature-time regimes

Germination temperature/°C	Germination rate/%*	*t*(germination)/h*					
24	48	72	24	48	72
*N*(germinated seeds)/%			*l*(sprout)/cm**		
24	(126.8±4.2)^b^	(63.2±3.4)^bC^	(75.8±1.6)^bB^	(80.0±1.2)^aA^	(0.23± 0.03)^cC^	(1.20±0.07)^cB^	(1.6±0.1)^cA^
28	(160.2±2.0)^a^	(85.2±0.4)^aB^	(88.0±1.0)^aA^	(93.0±1.0)^aA^	(0.7±0.1)^bC^	(1.7±0.2)^bB^	(2.5±0.2)^bA^
32	(161.7±1.8)^a^	(85.8±1.3)^aB^	(89.2±0.2)^aA^	(92.8±0.4)^aA^	(0.9±0.2)^aC^	(2.4±0.2)^aB^	(3.1±0.8)^aA^

Values are expressed as mean±standard deviation (*N*=5* and 10**). ^a-c^The values for germination temperature within a column are significantly different at p<0.05, ^A-C^The values for germination time within a row are significantly different at p<0.05

### Thousand kernel mass and germination losses

The total dry matter loss during germination can be expressed as a reduction in the thousand kernel mass. Thousand kernel mass gradually decreased as the germination progressed and higher reduction in it was at higher germination temperature due to increased germination loss (Table 2). Increased leaching loss at elevated temperature was due to higher grain diffusivity that resulted in higher solubilisation and leaching of galactomannans in the steep water (*15*). Higher metabolic and vegetative losses at elevated temperature and with the progression of germination time can be ascribed to increased enzymatic activity, as observed in the form of higher sprout length (Table 1). Furthermore, metabolic loss was higher than vegetative loss. Malleshi and Desikachar (*17*) also reported higher germination loss at increased germination temperature in malted barley. However, they reported higher vegetative loss than metabolic loss.

**Table 2:** Effect of germination temperature on thousand kernel mass and germination loss

Germination temperature/°C	*t*(germination)/h			*w*(leaching loss)/%	*t*(germination)/h								
24			48			72		
24	48	72			Germination loss/%						
*m*(thousand kernel)/g			Metabolic	Vegetative	Total	Metabolic	Vegetative	Total	Metabolic	Vegetative	Total
24	(6.68±0.04)^a^	(6.56± 0.10)^a^	(6.34±0.06)^a^	(1.89± 0.00)^c^	(1.57±0.04)^c^	(0.57±0.08)^c^	(4.04± 0.06)^c^	(2.7± 0.1)^c^	(1.23±0.09)^c^	(5.8±0.2)^c^	(3.94±0.08)^c^	(3.1±0.1)^c^	(8.9±0.1)^c^
28	(6.06±0.06)^b^	(5.57±0.09)^b^	(5.09±0.09)^b^	(2.28±0.05)^b^	(8.04± 0.09)^b^	(2.57±0.08)^b^	(12.9±0.1)^b^	(10.8±0.3)^b^	(5.9±0.2)^b^	(18.9±0.2)^b^	(13.2±0.1)^b^	(11.4±0.1)^b^	(27.0±0.1)^b^
32	(5.71±0.03)^c^	(5.12±0.06)^c^	(4.56±0.06)^c^	(2.94±0.05)^a^	(9.7± 0.1)^a^	(5.7±0.2)^a^	(18.4± 0.2)^a^	(12.1± 0.2)^a^	(11.4 ±0.1)^a^	(26.6±0.4)^a^	(17.0±0.1)^a^	(14.5±0.4)^a^	(34.6±0.8)^a^

*m*(thousand kernel)_initial_=6.95 g. Values are expressed as mean±standard deviation (*N*=5). Values with different letters in superscript within a column are significantly different at p<0.05

### Protein secondary structure

**Fig. S1:**
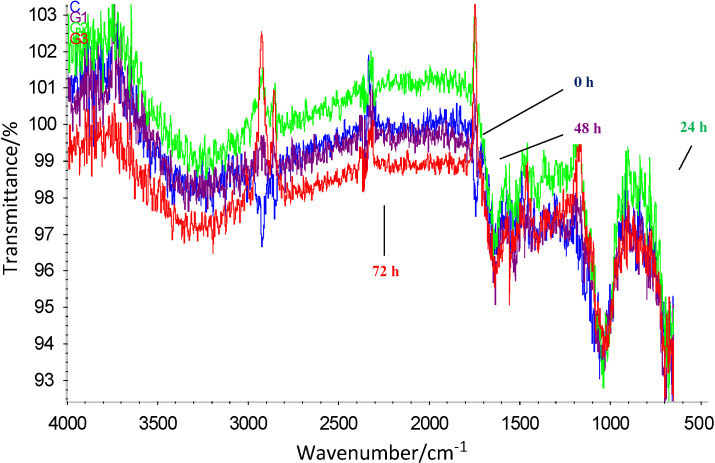
Effect of germination on the ATR-FTIR pattern of dhaincha flour

Changes in the secondary structure of proteins as a result of germination of dhaincha were observable in the peaks in the amide I and amide II regions at 1600-1700 cm^-1^ and 1580-1480 cm^-1^, respectively (*34*). Major peaks at 1662.3 (0 h), 1664.4 (24 h), 1667.1 (48 h) and 1676.2 (72 h) cm^-1^ exhibited conformation change in the secondary structure of the protein (Fig. S1). Shift in the peak from 1539.0 (0 h) to 1534.5 (24 h), 1535.7 (48 h) and 1533.6 (72 h) cm^-1^ and 1657.9 (0 h) to 1647.0 (24 h), 1646.4 (48 h) and 1647.7 (72 h) cm^-1^ can be associated with conformational alterations in the α-helix for amide II and I, respectively. Furthermore, the change in the peak from 1627.5 to 1635.1 cm^-1^ for flour germinated at 0 and 24 h respectively indicated mild conformational change in the β-sheet after germination for 24 h. However, the presence of unordered structure associated with peaks 1638.2 and 1639.3 cm^-1^ shows major conformational changes in the β-sheets after germination for 48 and 72 h, respectively.

### Technofunctional characteristics

Bulk density of the dhaincha flour decreased as the germination progressed (Table 3). Flour with reduced bulk density can be utilised for the formulation of weaning foods (*35*). Hydration properties of dhaincha flour are primarily a function of its galactomannans and proteins. The decline in the water absorption capacity after 48 h can be due to the utilisation of endosperm galactomannans as a substrate by germinating seed (*36*). However, an increase in the water absorption capacity after 72 h can be ascribed to denaturation of protein that resulted in the exposure of polar side chains and peptide bonds (*37*). Oil absorption capacity concomitantly increased as the germination proceeded due to exposure of hydrophobic residues as a result of protein unravelling, as validated by the FTIR spectra of germinated flour (Fig. S1). Elkhalifa and Bernhardt (*35*) reported a similar trend for the bulk density, and water and oil absorption capacity of germinated sorghum flour. Swelling index increased linearly with germination time due to weakened cell structure (*38*) that reduced the binding between the macromolecules (*19*). Swelling capacity is largely dictated by the degree of protein denaturation in flour since denatured proteins undergo amplified hydration in contrast to the native protein (*37*). Therefore, germination improved the swelling capacity of the dhaincha flour due to increased denaturation of the protein. Water solubility index and leaching loss increased up to 48 h due to depolymerisation and consequent leaching of galactomannans (*36*). However, slight reduction in the water solubility index and leaching loss after 72 h can be due to low protein solubility as a result of exposure of hydrophobic residues.

**Table 3:** Effect of germination at 28 °C on technofunctional characteristics of dhaincha flour

Functional property	*t*(germination)/h			
0	24	48	72
Bulk density/(g/cm^3^)	(0.5±0.0)^a^	(0.5±0.0)^b^	(0.5±0.0)^c^	(0.5±0.0)^c^
Water absorption capacity/(g/g)	(3.08±0.04)^a^	(2.40±0.04)^b^	(2.43±0.02)^b^	(3.03±0.03)^a^
Water solubility index/%	(15.3±0.6)^d^	(17.6±0.1)^c^	(22.0±0.5)^a^	(19.8±0.4)^b^
Oil absorption capacity/(g/g)	(0.92±0.03)^c^	(0.94±0.02)^bc^	(0.99±0.03)^b^	(1.03±0.03)^a^
Swelling index	(2.80±0.02)^d^	(3.10±0.02)^c^	(3.26±0.02)^b^	(3.80±0.02)^a^
Swelling capacity/(g/g)	(2.97±0.03)^d^	(3.24±0.01)^c^	(3.53±0.04)^b^	(3.63±0.02)^a^
Leaching loss/%	(24.0±0.3)^a^	(25.4±0.6)^ab^	(24.8±0.6)^b^	(22.2±0.0)^c^
Emulsification capacity/%	(50.0±0.0)^a^	(43.6±0.6)^b^	(44.9±0.8)^b^	(47.3±2.6)^ab^
Emulsion stability/%	(37.5±0.4)^a^	(25.6±0.5)^b^	(19.2±1.2)^c^	(14.1±0.6)^d^
Foaming capacity/%	(25.3±1.2)^d^	(53.3±1.2)^b^	(40.0±2.5)^c^	(120.0±2.0)^a^
Foam appearance	Dense foam	Moderately dense foam	Slightly dense foam	Foam with very large bubbles
Foam stability/%	(15.7±1.2)^bc^	(18.7±1.5)^b^	(14.0±2.0)^c^	(38.0±4.0)^a^
Dispersibility/%	(92.2±0.2)^a^	(92.7±0.8)^a^	(94.0±1.8)^a^	(93.5±1.0)^a^
Gel consistency in deionised water*/*cm	(14.22±0.04)^d^	(15.15±0.06)^b^	(15.65±0.02)^a^	(14.90±0.04)^c^
Gel consistency in acid/cm	(13.18±0.02)^d^	(15.70±0.04)^a^	(15.15±0.02)^b^	(15.84±0.03)^a^

Values are expressed as mean±standard deviation (*N*=3). The values within a row followed by different letters in superscript are significantly different at p<0.05.

Surface-active properties of dhaincha flour primarily depend on its protein fraction. Alteration in the emulsification and foaming capacity, as a result of germination was due to the denaturation of proteins and their consequent interaction with oil/water interface (*37*). Slight reduction in the emulsification capacity of germinated flour can be ascribed to conformational changes in the protein. However, a pronounced gradual reduction was observed in the emulsion stability with the increase in the germination time. Lower emulsion stability can be attributed to the aggregation of denatured proteins during heating of the emulsion, leading to clumping of the oil droplets. Foaming capacity of germinated flour was higher than of ungerminated (25.33%) and extremely high foaming capacity was observed after germination for 72 h (120%). Foaming capacity largely depends on the flexibility of the protein molecules where the ease of unravelling of polypeptide chain leads to high foaming capacity with large bubbles (*37*). Therefore, unordered structure caused by the unfolding of protein molecules and conformation changes in β-sheets as validated by the FTIR spectra (Fig. S1) can be ascribed to high foaming. Similar decline in the surface-active properties of black soybean was also observed after germination (*39*). The dispersibility of the flour showed no significant differences after germination. Germinated flour exhibited thinner consistency of gel in deionised water and acid than ungerminated flour due to depolymerisation of galactomannans in the germinated flour (*36*).

### Gelation characteristics

Heat-induced gelation of proteins involves the interaction of partially denatured proteins to form continuous three-dimensional network (*37*). However, the presence of a high amount of galactomannans can induce a detrimental effect on gelation by hampering the interaction of polypeptide chains with each other (*40*). However, the presence of galactomannans at low concentrations may induce better gelation (*41*). Therefore, the gelation behaviour of germinated dhaincha flour is a cumulative function of depolymerisation of galactomannans and conformation changes in proteins, and their relative interaction during gel formation. Ungerminated dhaincha flour exhibited the lowest gelation (for full gelation 20% flour was needed), whereas all the germinated flour samples reached full gelation at 18% flour (Table 4). Firm gel was observed at 30% flour in all the samples. However, variation in the gel appearance was observed at lower flour contents. Particularly, the presence of clotted particles at low flour contents in germinated flour (48 and 72 h) can be a result of the protein aggregation due to hydrophobic interactions. Sharma and Sahni (*42*) observed the clotted particles at low content (2-6%) of lucerne flour and for full gelation only 8% of germinated lucerne flour was necessary.

**Table 4:** Effect of germination at 28 °C on gelation behaviour of dhaincha flour

*t*(germination)/h	Property	(*m*(flour)/*V*(water))/%																										
2	4	6		8	10		12		14						16		18		20		22		25		30	
0	Gelation	-	-	-		-	-		-		+						+		+		+		+		+		+	
	Appearance	L	L	L		L	L		L			V					C		C		G		G		G		FG	
24	Gelation	-	-	-		-	-		-		+						+		+		+		+		+		+	
	Appearance	L	L	L		L	L		L					V			C		G		G		G		G		FG	
48	Gelation	-	-	-		-	-		-					-			-		+		+		+		+		+	
	Appearance	L	L	L		LWCP		LWCP		LWCP					LWCP			LWCP		G		G		G		G		FG	
72	Gelation	-	-	-		-	-		-		+					+			+		+		+		+		+	
	Appearance	L	L	L	LWCP		LWCP		LWCP				C			C			G		G		G		G		FG	

L=liquid, V=viscous, C=curdy, G=gel, F=firm gel, LWCP=liquid with clotted particles, + gelation, ± partial gelation, – no gelation

### Antinutrients

Germination resulted in 24.47% reduction in the tannin mass fraction (Table 5). Initial soaking period resulted in leaching of water-soluble tannins in steep water, whereas the reduction in tannins during germination was primarily due to oxidation (*6*). Phytic acid mass fraction gradually decreased as the germination progressed and 16.38% reduction in the phytic acid mass fraction was observed after 72 h. The reduction of phytic acid mass fraction during germination was due to leaching in steep water (*43*) and endogenous phytase activity (*3*). Sahni *et al.* (*43*) reported 13.90% reduction in the phytic acid content in dhaincha after soaking. Similar trend was also observed for saponins and 24.58% reduction was observed after germination (72 h). The reduction in the saponins is caused by their leaching during the soaking as well as remobilisation and translocation of saponins to sprout fraction of the seed during germination (*44*). Trypsin inhibitor and lectin activity were reduced by 40.33 and 62.5% respectively after germination for 72 h, due to their inactivation as a result of alternation in their structures (*45*). Duhan *et al.* (*5*) reported similar trend in the reduction of saponin and trypsin inhibitor activity in germinated pigeon pea.

**Table 5:** Effect of germination at 28 °C on biofunctional characteristics of dhaincha flour

*t*(germination)/h	Antinutrient				Bioactive constituent			Antioxidant activity				
*w*(tannins)/(mg/g)	*w*(phytic acid)/(mg/g)	*w*(saponins as DE)/(mg/g)	Trypsin inhibitor activity/ (IU per mg protein)	Lectin activity (as haemagglutinin)/(U/g)	*w*(total phenolics as GAE)/(mg/g)	*w*(total flavonoids as QE)/(mg/g)	*b*(DPPH˙ as TE)/(µmol/100 g)	*b*(ABTS˙^+^ as TE)/(µmol/100 g)	*b*(FRAP as TE)/(µmol/g)	Reducing power as AAE/(mg/g)	*b*(EDTAE)/(mmol/100 g)
0	(22.72±0.05)^a^	(13.73±0.04)^a^	(3.01±0.02)^a^	(54.2±1.3)^a^	400^a^	(21.6±0.6)^a^	(0.94±0.02)^d^	(157.50±0.04)^a^	(159.8±1.0)^e^	(34.89±0.08)^e^	(12.23±0.06)^a^	(2.69±0.04)^b^
24	(18.80±0.07)^b^ (-17.16)	(12.75±0.03)^b^ (-7.13)	(2.58±0.02)^b^ (-14.28)	(48.5±0.9)^b^ (-10.47)	400^a^	(13.8±0.2)^d^ (-35.99)	(1.03±0.01)^c^ (+9.57)	(156.0±0.0)^b^ (- 1.5)	(161.3±1.2)^a^	(21.4±0.1)^d^ (- 38.60)	(7.79±0.01)^d^ (-36.30)	(3.39±0.05)^a^ (+26.02)
48	(17.55 ±0.03)^c^ (-22.75)	(12.02±0.06)^c^ (-11.07)	(2.39±0.04)^c^ (-23.25)	(39.3±0.6)^c^ (-27.34)	200^b^ (-50)	(14.4±0.3)^c^ (-33.11)	(1.23±0.01)^b^ (+30.85)	(154.5±0.0)^c^ (- 3.0)	(148.1±1.0)^b^ (-11.7)	(23.8±0.1)^c^ (- 31.78)	(8.1±0.0)^c^ (-33.85)	(3.35±0.06)^a^ (+24.53)
72	(17.16±0.03)^d^ (-24.47)	(11.48±0.04)^d^ (-16.38)	(2.27±0.02)^d^ (-24.58)	(32.31±1.66)^d^ (-40.33)	150^b^ (-62.5)	(15.46±0.62)^b^ (-28.29)	(1.28±0.02)^a^ (+36.14)	(153.8±0.0)^d^ (-3.7)	(141.3±0.8)^c^ (-18.5)	(28.04±0.04)^b^ (-19.63)	(8.66±0.05)^b^ (-29.19)	(3.41±0.05)^a^ (+26.76)

Values are expressed as mean±standard deviation (*N*=3) at *w*(moisture)=8%. The values within a column with different superscripts are significantly different at p<0.05. Values in the parentheses represent the percentage of change, where (-) and (+) signs represent a decrease and increase, respectively, in comparison with the unprocessed sample. IU=inhibition unit, GAE=gallic acid equivalents, QE=quercetin equivalents, TE= Trolox equivalents, AAE=ascorbic acid equivalents, EDTAE=ethylenediamine tetraacetic acid equivalents

### Biofunctional characteristics

The content of total phenolics of germinated flour was lower than of ungerminated flour. The lowest total phenolic mass fraction was observed after 24 h, followed by a slight increase as the germination progressed. The comparatively lower mass fraction of total phenols after 24 h can be due to their loss during soaking in the steep water. Contrary to total phenols, the mass fraction of total flavonoids gradually increased with the increase in the germination period. The phenol and flavonoid mass fractions increased after germination due to their biogenesis, and as the result of release of bound phenols from the weakened cell wall (*46*, *47*). Bubelová *et al.* (*48*) also reported an increase in the flavonoid content of lentils after germination.

**Fig. 2:**
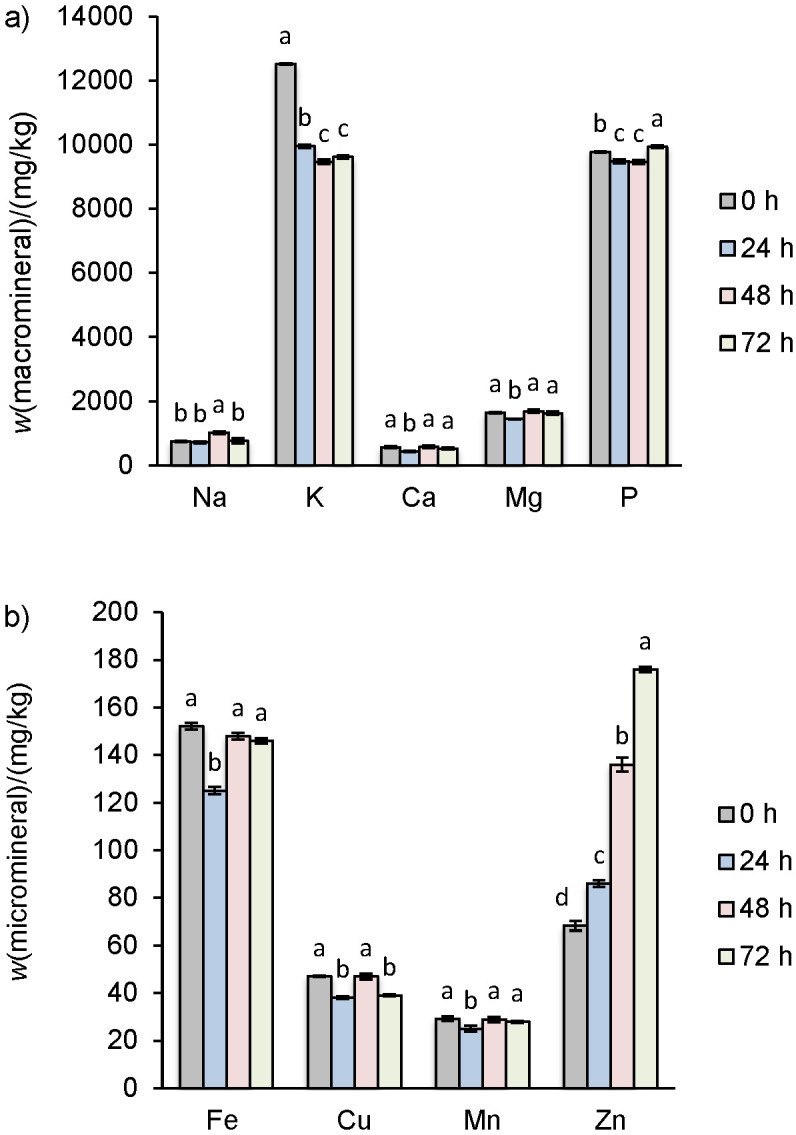
Effect of germination time on the mineral profile: a) macrominerals, and b) microminerals of dhaincha flour. Values are expressed as mean and error bars represent standard deviation (*N*=3) at 8% moisture content. The mean values with different letters are significantly different at p<0.05

Germination can result in loss/biogenesis of various antioxidant species that affect the antioxidant capacity of germinated flour. Furthermore, antioxidant species formed during germination also exhibited variability in their affinity towards different prooxidant species. Therefore, different methods were employed to elucidate the effect of germination on the antioxidant capacity of dhaincha. DPPH˙ and ABTS˙^+^ scavenging activities gradually decreased with the progression of germination. The decrease with time of DPPH˙ scavenging activity expressed as TE (157.5 to 153.8 µmol/100 g) was lower than of ABTS˙^+^ scavenging activity (159.8 to 141.3 µmol/100 g). However, high positive correlation was observed (R=0.90) between the DPPH˙ and ABTS˙^+^ scavenging activity. Maximum decrease in Fe(III) ion reducing antioxidant power and reducing power was observed after 24 h, followed by a gradual enhancement after germination for 48 and 72 h. Decrease in the total phenolics showed a high positive correlation with the resultant decrease in FRAP (R=0.96) and reducing power (R=1.00). Metal chelating activity increased (expressed as EDTA, 2.69 to 3.41 mmol/100 g) with the germination and showed a positive correlation (R=0.74) with the increase of flavonoid mass fraction and negative correlation (R=-0.850) with the phytic acid mass fraction. Phytic acid, as phytate, forms strong complexes with many metal ions, thus competing in complexation with EDTA. Increase in the metal chelating activity can be attributed to the increase in the availability of metal ions (Fig. 2) due to reduction in phytic acid mass fraction (Table 5). Al-Laith *et al.* (*49*) and Liu *et al.* (*50*) also documented a high correlation of Fe(III) ion reducing antioxidant power and reducing power with total phenolics and their comparatively much weaker correlation with DPPH˙ and ABTS˙^+^ scavenging activity.

### Macro- and microminerals

During germination, various minerals may be lost or their mass fraction increased as a result of leaching, solid loss, modification of cell structure, reduction of antinutrients and mineral remobilisation (*42*). Na mass fraction was slightly higher after 48 h, whereas the mass fraction of Ca, Mg, Fe and Mn was the lowest after 24 h (Fig. 2). Furthermore, P and Mn mass fractions also decreased after 24 h. The reduction in the mineral mass fraction after 24 h can be explained with the loss of minerals in the steep water during soaking as well as remobilisation to the sprouting part of the seed. In addition, minerals are also utilised in various metabolic processes by seeds during germination (*51*). However, an increase in the mineral mass fraction on further germination can be ascribed to the decrease in the phytic acid and tannin content that tend to bind minerals (*52*). Furthermore, germination causes degradation of the cellular structure by cell wall-degrading enzymes that can result in better extraction of minerals (*47*). Germination caused drastic increase in the Zn mass fraction. Zn is predominantly present in the cotyledon of legumes (*53*) and tends to interact with proteins as well as other macromolecules and reduce their extractability (*54*). Germination causes modification of cellular structure of the grain by forming microcracks and fissures and weakening the bond of Zn with other macromolecules, consequently improving their extractability (*38*). Sharma and Sahni (*42*) reported increased mass fraction of Zn in germinated lucerne flour in comparison with the ungerminated counterpart.

### Principal component analysis

**Fig. 3:**
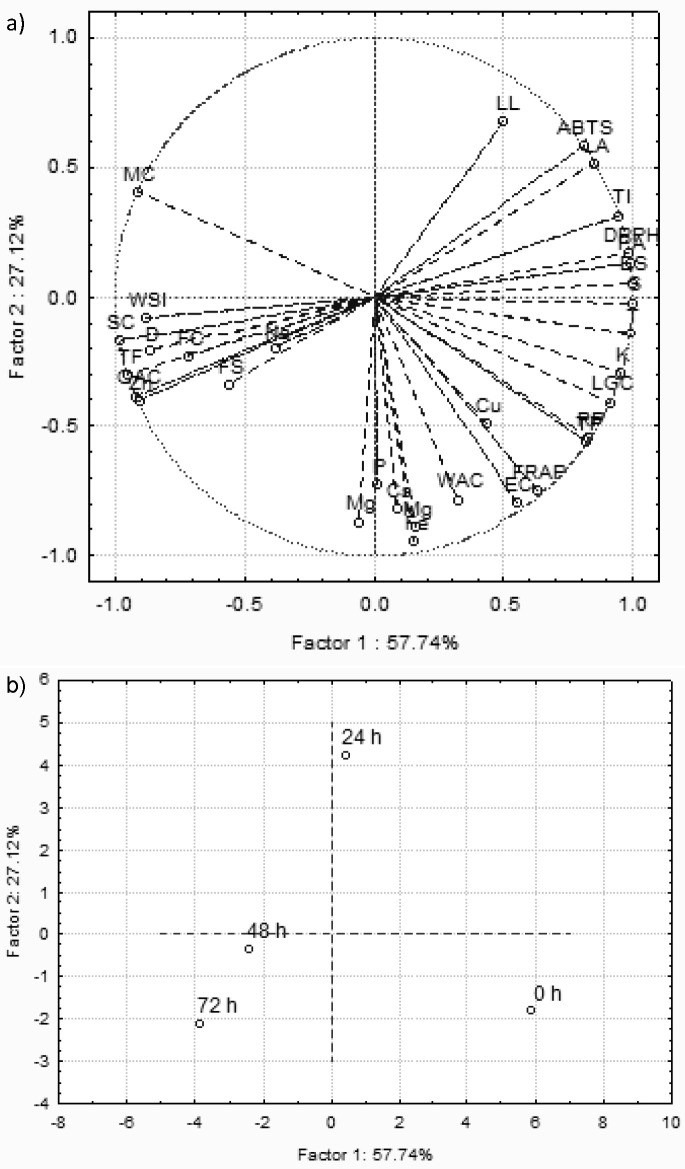
Principal component analysis (PCA) showing: a) loading, and b) score plot describing the relationship between the germination and characteristics of dhaincha flour. WAC=water absorption capacity, WSI=water solubility index, OAC=oil absorption capacity, SC=swelling capacity, LL=leaching loss, EC=emulsification capacity, ES=emulsion stability, FC=foaming capacity, FS=foam stability, D=dispersibility, LGC=lowest gelation concentration, T=tannins, PA=phytic acid, S=saponins, TI=trypsin inhibitor, LA=lectin activity, TP=total phenolic content, TF=total flavonoids, DPPH RSA=DPPH radical scavenging activity, ABTS RSA=ABTS radical scavenging activity, FRAP=ferric reducing antioxidant power, RP=reducing power, MC=metal chelating activity, macrominerals: Na, K, Ca, Mg and P, microminerals: Fe, Cu, Mn and Zn

PCA loading plot (Fig. 3a) shows the relationship between germination and characteristics of dhaincha flour samples. Positive correlation was observed between the total phenolic content and ferric reducing antioxidant power and reducing power, whereas they were not correlated with DPPH˙ and ABTS˙^+^ scavenging activity, which showed a correlation between them. PCA score plot (Fig. 3b) reflects demarcation in the characteristics of germinated dhaincha flour compared to ungerminated flour, and also reflected variation amongst germinated samples. However, samples germinated for 48 and 72 h were more closely related to each other and reflected negative correlation with antinutrients and positive correlation with swelling and foaming capacities, foam stability, total flavonoids and Zn. The magnitude of the aforesaid changes was higher in the sample germinated for 72 h. Overall, germination for 72 h resulted in a higher reduction of antinutrients with comparatively smaller compromise of antioxidant activity and exhibited better functional characteristics.

## CONCLUSIONS

The most suitable temperature for sprouting dhaincha seeds owing to high germination capacity and comparatively lower germination loss was 28 °C. The regime of 28 °C for 72 h was optimum for the germination of dhaincha. Under these conditions, the maximum reduction of antinutrients was obtained with optimal preservation of antioxidant capacity and drastic improvement in the extractability of Zn and flavonoids. Furthermore, germination (72 h) markedly enhanced the hydration and foaming capacity, which allow wider utilisation of germinated dhaincha flour for the development of food products. The utilisation of germinated dhaincha flour is particularly recommended for the development of pasta and baked product based on the improved functionality of flour.
